# Comparative Effectiveness of Intracranial Pressure Monitoring vs No Monitoring in Severe Penetrating Brain Injury Management

**DOI:** 10.1001/jamanetworkopen.2023.1077

**Published:** 2023-03-24

**Authors:** Ali Mansour, Susan Rowell, Plamena P. Powla, Peleg Horowitz, Fernando D. Goldenberg, Christos Lazaridis

**Affiliations:** 1 Division of Neurocritical Care, Department of Neurology, The University of Chicago Medical Center, Chicago, Illinois; 2 Department of Neurosurgery, The University of Chicago Medical Center, Chicago, Illinois; 3 Division of Trauma and Acute Care Surgery, Department of Surgery, The University of Chicago Medical Center, Chicago, Illinois

## Abstract

**Importance:**

Civilian penetrating brain injury (PBI) is associated with high mortality. However, scant literature is available to guide neurocritical care monitoring and management of PBI.

**Objective:**

To examine the association of intracranial pressure (ICP) monitoring with mortality, intensive care unit (ICU) length of stay (LOS), and dispositional outcomes in patients with severe PBI.

**Design, Setting, and Participants:**

This comparative effectiveness research study analyzed data from the Trauma Quality Improvement Program of the National Trauma Data Bank in the US from January 1, 2017, to December 31, 2019. Patients with PBI were identified, and those aged 16 and 60 years who met these inclusion criteria were included: ICU LOS of more than 2 days, Glasgow Coma Scale (GCS) score lower than 9 on arrival and at 24 hours, and Abbreviated Injury Scale score of 3 to 5 for the head region and lower than 3 for other body regions. Patients with bilaterally fixed pupils or incomplete data were excluded. A 1:1 propensity score (PS) matching was used to create a subgroup of patients. Patients were divided into 2 groups: with vs without ICP monitoring. Data analysis was conducted between September and December 2022.

**Exposures:**

Intracranial pressure monitoring vs no monitoring.

**Main Outcomes and Measures:**

Outcomes were mortality, rate of withdrawal, ICU LOS, and dispositional outcome. Measures were age, initial systolic blood pressure, initial oxygen saturation level on a pulse oximeter, first-recorded GCS score, GCS score at 24 hours, Abbreviated Injury Scale score, midline shift, and pupillary reactivity.

**Results:**

A total of 596 patients (505 males [84.7%]; mean [SD] age, 32.2 [12.3] years) were included, among whom 220 (36.9%) died and 288 (48.3%) had ICP monitoring. The PS matching yielded 466 patients (233 in each group with vs without ICP monitoring). Overall mortality was 35.8%; 72 patients with ICP monitoring (30.9%) died compared with 95 patients (40.8%) without ICP monitoring . Patients with ICP monitoring were more likely to survive (odds ratio [OR], 1.54; 95% CI, 1.05-2.25; *P* = .03; number needed to treat, 10). No difference in favorable discharge disposition was observed. The PS-weighted analysis included all 596 patients and found that patients with ICP monitoring were more likely to survive than those without (OR, 1.40; 95% CI, 1.10-1.78; *P* = .005). The E-value for the OR calculated from the PS-matched data set was 1.79. In addition, ICP monitoring vs no monitoring was associated with an increase in median (IQR) ICU LOS (15.0 [8.0-21.0] days vs 7.0 [4.0-12.0] days; *P* < .001).

**Conclusions and Relevance:**

In this comparative effectiveness research study, PBI management guided by ICP monitoring was associated with decreased mortality and increased ICU LOS, challenging the notion of universally poor outcomes after civilian PBI. Randomized clinical trials that evaluate the efficacy of ICP monitoring in PBI are warranted.

## Key Points

**Question:**

Is intracranial pressure (ICP) monitoring effective in the management of severe penetrating brain injury (PBI)?

**Findings:**

In this comparative effectiveness research study involving 596 patients, ICP monitoring was associated with decreased mortality and increased length of stay in the intensive care unit among patients with severe PBI.

**Meaning:**

Findings of this study challenge the notion of universally poor outcomes after civilian PBI and may warrant future randomized clinical trials that evaluate the efficacy of ICP monitoring in PBI.

## Teaser

This comparative effectiveness research study assesses the mortality rate, life-sustaining treatment withdrawal rate, length of intensive care unit stay, and discharge disposition in patients with penetrating brain injury (PBI)with or without intracranial pressure (ICP) monitoring.

## Introduction

More than 48 000 firearm-related deaths occurred in the US in 2021, the highest reported number since 1994.^1,2^ Civilian penetrating brain injury (PBI) primarily affects those younger than 40 years, with mortality rates of 40% to 90%.^1,2^ A study based on the National Sample Program of the National Trauma Data Bank (NTDB) that included over 8000 adults with firearm-inflicted head injuries between 2003 and 2012 reported a mean patient age of 36 years with a 55% mortality rate.^3^ Another study using the Trauma Quality Improvement Program (TQIP) database, which included over 26 000 patients with PBI from 2010 to 2014, also found a mean patient age of 36 years and a mortality rate of 44%.^4^ Despite the high prevalence and likelihood of morbid outcomes associated with PBI, the body of knowledge pertaining to management and prognosis of PBI has received little contribution since the inception of the Guidelines for the Management of Penetrating Brain Injury in 2001.^5,6^ Given that PBI is an exclusion criterion in most randomized clinical trials (RCTs) of severe traumatic brain injury (TBI), the generalizability of knowledge from blunt to penetrating injuries is limited.^7^

Intracranial pressure (ICP) monitoring and ICP-guided management are a cornerstone in the approach to blunt severe TBI.^8^ Intracranial hypertension has been associated with mortality in observational cohorts.^9,10,11,12^ Additionally, several studies have suggested that care in specialized centers that practice protocol-driven therapy—typically including ICP monitoring—is associated with lower mortality and better outcomes in patients with severe TBI.^13,14,15,16,17,18^ Reports of benefit are not universal, however, as several studies have found either no benefit or harm from ICP-guided management.^19,20,21,22,23^ A single RCT conducted in Bolivia and Ecuador compared ICP monitoring with no monitoring in the management of severe TBI and reported no significant between-group difference in morbidity or mortality measured at 6 months postinjury.^24^

In view of these findings, the latest iteration of the Brain Trauma Foundation guidelines issued a level IIb recommendation (low-quality evidence) of ICP monitoring to reduce in-hospital and 2-week postinjury mortality.^25^ This recommendation is based solely on reports for blunt TBI, as there are insufficient data available to support a treatment standard for the use of ICP monitoring in PBI.^26^ Given the paucity of evidence, we extracted data from the NTDB to conduct a propensity score (PS)–matched comparative effectiveness research study of PBI management guided by monitoring vs no monitoring.^27^ We aimed to examine the association of ICP monitoring with mortality, intensive care unit (ICU) length of stay (LOS), and dispositional outcomes in patients with severe PBI.

## Methods

### Study Design

This observational comparative effectiveness research study used data from the TQIP of the NTDB, a prospective, curated trauma registry with the purpose of tracking and providing information about trauma care and outcomes. The NTDB is the world’s largest trauma data repository, with more than 7.5 million electronic records from more than 900 trauma centers in the US.^27^ The University of Chicago Institutional Review Board deemed this study exempt from institutional review board review and waived the informed consent requirement because the study was a retrospective analysis of a fully deidentified database. We followed the Strengthening the Reporting of Observational Studies in Epidemiology (STROBE) and the International Society for Pharmacoeconomics and Outcomes Research (ISPOR) reporting guidelines. Furthermore, in accordance with the ISPOR reporting guideline for comparative effectiveness research, we performed a sensitivity analysis to estimate the level of confounding that would have driven the results back toward the null hypothesis.^28^

**Figure 1.**
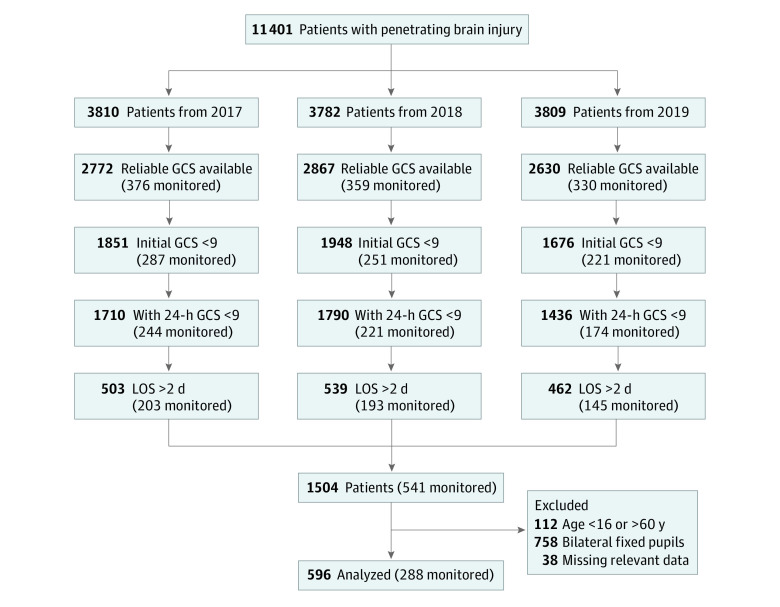
Flowchart Cohort Selection GCS indicates Glasgow Coma Scale; LOS, length of stay; monitored, with intracranial pressure monitoring.

Data from the NTDB for January 1, 2017, through December 31, 2019, were surveyed. This 3-year period was chosen to include a contemporary data set, including the data obtained after the Brain Trauma Foundation guidelines were updated in 2017, and to minimize missing key variables, such as pupillary reactivity and midline shift occurring in prior years. We identified 11 401 cases of PBI using *International Statistical Classification of Diseases and Related Health Problems, Tenth Revision,* codes (eTable in Supplement 1) with data on a concomitant mechanism of injury that was penetrating from trauma centers participating in the TQIP. From this population, 1504 patients met the following inclusion criteria: ICU LOS of more than 2 days, Glasgow Coma Scale (GCS) score lower than 9 on arrival and at 24 hours (score range: 3-8), and Abbreviated Injury Scale (AIS) score of 3 to 5 for the head region and lower than 3 for other body regions. Patients between the ages of 16 and 60 years were included (n = 1392). Patients with bilaterally fixed pupils were excluded, leaving 634 observations. Patients with incomplete or erroneous data on the intervention of interest (ICP monitoring status), outcomes of interest (eg, hospital disposition, ICU LOS, and withdrawal of life-sustaining therapy [WOLST]), and variables of interest (eg, systolic blood pressure [SBP; excluded if <20 mm Hg], oxygen saturation level [excluded if <20%] on a pulse oximeter, GCS score, and data on midline shift and pupillary reactivity) were also excluded, yielding a data set with 596 patients (Table 1 and Figure 1). Of these patients, 288 (48.3%) had an ICP monitor placed.

**Table 1.** Characteristics of Patients With Penetrating Brain Injury From 2017 to 2019

Variable	No. (%)		
	Overall	ICP monitoring	
		Without	With
No. of patients	596 (100)	308 (51.7)	288 (48.3)
Age, mean (SD), y	32.2 (12.3)	32.8 (12.3)	31.6 (12.2)
Systolic BP, mean (SD), mm Hg	130.6 (32.1)	128.1 (33.0)	133.2 (31.1)
Heart rate, mean (SD), bpm	98.6 (29.3)	100.0 (30.3)	97.1 (28.2)
Oxygen saturation level on pulse oximeter, mean (SD), %	97.1 (6.6)	96.8 (7.1)	97.4 (6.2)
ISS, median (IQR)	25.0 (20.0-29.0)	25.0 (20.0-29.0)	25.0 (20.0-29.0)
Sex			
Female	91 (15.3)	47 (15.3)	44 (15.3)
Male	505 (84.7)	261 (84.7)	244 (84.7)
Total GCS score on arrival			
3	361 (60.6)	201 (65.3)	160 (55.6)
4	41 (6.9)	14 (4.5)	27 (9.4)
5	32 (5.4)	17 (5.5)	15 (5.2)
6	55 (9.2)	25 (8.1)	30 (10.4)
7	63 (10.6)	29 (9.4)	34 (11.8)
8	44 (7.4)	22 (7.1)	22 (7.6)
Total GCS score at 24 h			
3	221 (37.1)	130 (42.2)	91 (31.6)
4	40 (6.7)	21 (6.8)	19 (6.6)
5	32 (5.4)	24 (7.8)	8 (2.8)
6	83 (13.9)	34 (11.0)	49 (17.0)
7	133 (22.3)	55 (17.9)	78 (27.1)
8	87 (14.6)	44 (14.3)	43 (14.9)
Pupillary reactivity			
1 Pupil reactive	152 (25.5)	85 (27.6)	67 (23.3)
Both pupils reactive	444 (74.5)	223 (72.4)	221 (76.7)
No midline shift	359 (60.2)	199 (64.6)	160 (55.6)
Firearm-related injury	558 (93.6)	286 (92.9)	272 (94.4)
Year of data			
2017	201 (33.7)	98 (31.8)	103 (35.8)
2018	212 (35.6)	111 (36.0)	101 (35.1)
2019	183 (30.7)	99 (32.1)	84 (29.2)
ICU LOS, median (IQR), d	10.0 (5.0-17.0)	6.0 (4.0-12.0)	15.0 (8.0-21.0)
Unfavorable outcome	350 (58.7)	185 (60.1)	165 (57.3)
WOLST	124 (20.8)	74 (24.0)	50 (17.4)
Mortality	220 (36.9)	135 (43.8)	85 (29.5)

Abbreviations: BP, blood pressure; bpm, beats per minute; GCS, Glasgow Coma Scale (score range: 3-8); ICP, intracranial pressure; ICU LOS, intensive care unit length of stay; ISS, Injury Severity Score; WOLST, withdrawal of life-sustaining therapy.

### Neurological Factors and Outcome Variables Definition

Intracranial pressure monitoring was defined as the presence of an intraparenchymal monitor or an external ventricular drain. Variables of interest were the following: age, initial SBP, initial oxygen saturation level, first recorded GCS score within 30 minutes or less of arrival, GCS score at 24 hours (defined as the highest total GCS score on calendar day after arrival to index hospital), AIS score, midline shift (defined as >5 mm within 24 hours of injury), and pupillary reactivity at arrival. This variable selection follows the recommendations by Haider et al^29^ when studying mortality and includes neurological factors, such as pupillary reactivity, midline shift, and highest GCS score in the first 24 hours.

Outcomes of interest were mortality, rate of WOLST, ICU LOS, and dispositional outcome as defined by the hospital discharge location. Mortality was defined as death by the time of hospital discharge or discharge to hospice.^30^ The NTDB does not include data on costs; laboratory values; and long-term outcomes, including readmissions and functional outcomes. For that reason, we elected to define unfavorable dispositional outcome as death or discharge to a hospice facility, a long-term acute care hospital, or a skilled nursing facility. Favorable dispositional outcomes included leaving against medical advice; transfer to court, law enforcement, or a psychiatric hospital; or discharge to home, a short-term or intermediate care hospital, or an acute rehabilitation facility.

### Statistical Analysis

Data analysis was conducted between September and December 2022. The data set was divided into 2 groups: patients with or patients without an ICP monitor. Statistical analyses were conducted using parametric unpaired, 2-tailed *t* test for continuous data and Pearson χ^2^ test for categorical data. Complete information on all variables was available for 596 patients. Standardized mean difference (SMD) was used to examine the balance of covariate distribution between the 2 groups (with or without ICP monitoring). A 2-sided *P* < .05 was considered to be statistically significant.

We used PS matching to create a subgroup of patients among whom the covariates were distributed in a balanced fashion and to reduce bias in estimates of treatment effect.^31,32^ Logistic regression was performed to define the probability of receiving an ICP monitor. Variables used in the PS analysis included age, initial GCS score, initial SBP, GCS score at 24 hours, presence of midline shift on head computed tomography, Injury Severity Score, pupillary reactivity, and WOLST rate. Distribution of variables and SMD results for the matched cohort are presented in Table 2.

**Table 2.** Characteristics of Propensity Score–Matched Patients With Penetrating Brain Injury From 2017 to 2019

Variable	No. (%)			*P* value	SMD
	Overall	ICP monitoring			
		Without	With		
No. of patients	466 (100)	233 (50.0)	233 (50.0)	NA	NA
Age, mean (SD), y	32.3 (12.2)	32.3 (12.2)	32.2 (12.2)	.93	.01
Systolic BP, mean (SD), mm Hg	131.7 (32.2)	132.2 (33.7)	131.2 (30.7)	.74	.03
Heart rate, mean (SD), bpm	99.2 (29.5)	99.4 (30.8)	99.0 (28.2)	.88	.01
Oxygen saturation level on pulse oximeter, mean (SD), %	97.2 (6.6)	97.0 (6.7)	97.3 (6.6)	.64	.04
ISS, median (IQR)	25.0 (20.0-29.0)	25.0 (20.0-29.0)	25.0 (20.0-29.0)	.41	.01
Sex					
Male	397 (85.2)	199 (85.4)	198 (85.0)	.97	.05
Female	69 (14.8)	34 (14.6)	35 (15.0)		
Total GCS score on arrival					
3	280 (60.1)	143 (61.4)	137 (58.8)	.78	.15
4	31 (6.7)	12 (5.2)	19 (8.2)		
5	21 (4.5)	10 (4.3)	11 (4.7)		
6	48 (10.3)	24 (10.3)	24 (10.3)		
7	51 (10.9)	28 (12.0)	23 (9.9)		
8	35 (7.5)	16 (6.9)	19 (8.2)		
Total GCS score at 24 h					
3	172 (36.9)	85 (36.5)	87 (37.3)	.97	.09
4	33 (7.1)	16 (6.9)	17 (7.3)		
5	20 (4.3)	12 (5.2)	8 (3.4)		
6	62 (13.3)	31 (13.3)	31 (13.3)		
7	108 (23.2)	54 (23.2)	54 (23.2)		
8	71 (15.2)	35 (15.0)	36 (15.5)		
Pupillary reactivity				.67	.05
1 Pupil reactive	123 (26.4)	64 (27.5)	59 (25.3)		
Both pupils reactive	343 (73.6)	169 (72.5)	174 (74.7)		
No midline shift	284 (60.9)	138 (59.2)	146 (62.7)	.51	.07
Firearm-related injury	432 (92.7)	214 (91.8)	218 (93.6)	.59	.07
Year of data					
2017	157 (33.7)	76 (32.6)	81 (34.8)	NA	NA
2018	160 (34.3)	79 (33.9)	81 (34.8)		
2019	149 (32.0)	78 (33.5)	71 (30.5)		
ICU LOS, median (IQR), d	10.0 (5.0-17.0)	7.0 (4.0-12.0)	15.0 (8.0-21.0)	<.001	NA
Unfavorable outcome	266 (57.1)	135 (57.9)	131 (56.2)	.78	NA
WOLST	94 (20.2)	48 (20.6)	46 (19.7)	.91	.02
Mortality	167 (35.8)	95 (40.8)	72 (30.9)	.03	NA

Abbreviations: BP, blood pressure; bpm, beats per minute; GCS, Glasgow Coma Scale (score range: 3-8); ICP, intracranial pressure; ICU LOS, intensive care unit length of stay; ISS, Injury Severity Score; NA, not applicable; SMD, standardized mean difference; WOLST, withdrawal of life-sustaining therapy.

After PS calculation using logistic regression, we used the MatchIt library in R, version 2022.07.2 (RStudio) and full matching, nearest neighbor matching, and exact matching. Using the logistic regression method to calculate PS followed by a nearest neighbor matching approach yielded favorable matching as suggested by SMD testing in the matched cohort (Table 2). Matching was performed on a 1:1 basis. The final association of ICP monitoring with mortality, ICU LOS, and dispositional outcome was calculated using the matched cohort via a simple logistic regression with 233 patients in each group (with or without ICP monitoring).

In a separate analysis that included the original data set (596 patients), we used PS that was calculated from the data set to derive inverse probability PS weights, with inverse probability weighting = 1/PS and 1/(1 − PS), for patients with vs without ICP monitoring, respectively. Inverse probability PS weights were introduced into a weighted multiple logistic regression model examining the association between ICP monitoring status and the outcome of interest.

Sensitivity analysis was performed to assess for potential unmeasured or uncontrolled confounding and to determine the robustness of the association between ICP monitoring and mortality.^33^ The E-value was defined as the minimum strength of association needed between an unmeasured confounder and both ICP monitoring and the outcome to fully explain the specific association between treatment and outcome, depending on the measured covariates. The sensitivity analysis was used to assess for hidden bias that could have affected the results of the PS-matched analysis.

## Results

Of the 11 401 patients with PBI identified from the NTDB during the study period (3810 from 2017, 3782 from 2018, and 3809 from 2019), 1504 met the inclusion criteria and 596 were included in the target population (Figure 1). These patients consisted of 505 males (84.7%) and 91 females (15.3%) with a mean (SD) age of 32.2 (12.3) years. Two hundred twenty patients (36.9%) died. Two hundred eighty-eight patients (48.3%) had an ICP monitor, with a median (IQR) time to placement of 1 [1-1] day. Table 1 and Table 2 provide the number of patients and distribution of outcomes.

Across the 3-year study period, ICP monitor use rates were relatively similar (51.2% in 2017, 47.6% in 2018, and 45.9% in 2019; *P* = .56). The mechanism of PBI was firearm-related in 558 patients (93.6%), with the remaining cases attributed to low-velocity mechanisms. The GCS score distribution at 24 hours was different between the 2 groups, with a higher percentage of patients without ICP monitoring having a GCS score of 3 compared with patients with ICP monitoring (42.2% [130 of 308 patients] vs 31.6% [91 of 288 patients]; *P* = .001) (Table 1). Patients without ICP monitoring were less likely to have a midline shift on head computed tomography than patients with ICP monitoring (64.6% [199 patients] vs 55.6% [160 patients]; *P* = .03) (Table 1).

**Figure 2.**
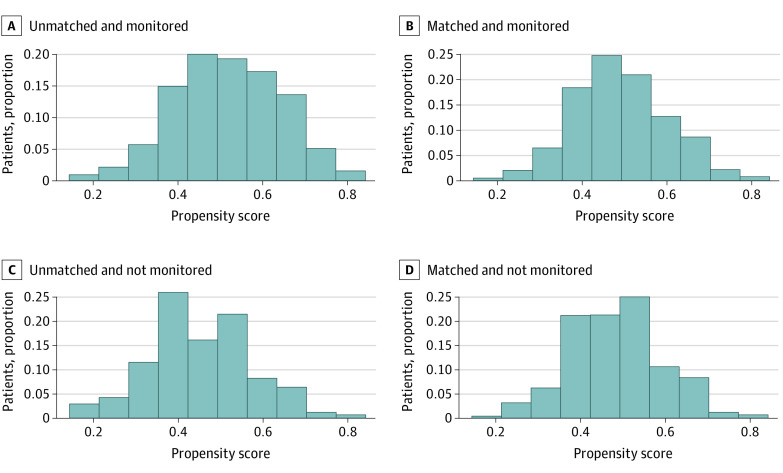
Distribution of Propensity Scores Before and After Matching

Of all of the patients included in the analysis, 466 were PS-matched (233 in each group). Figure 2 shows the PS distribution before and after matching. Table 2 shows the SMD for the variables of interest in the matched cohort. All variables except for GCS score on arrival were within less than 0.1 SMD of each other (SMD <0.1 was considered to be negligible).^31^

In the PS-matched cohort, with an overall mortality of 35.8%, 72 patients (30.9%) with ICP monitoring died compared with 95 patients (40.8%) without monitoring. Patients with ICP monitoring were more likely to survive (odds ratio [OR], 1.54; 95% CI, 1.05-2.25; *P* = .03; number needed to treat, 10). No difference in favorable discharge disposition was observed between groups (OR, 1.07; 95% CI, 0.57-1.55; *P* = .71). Similar to the 1:1 PS-matched cohort analysis, the inverse probability PS–weighted analysis that included all 596 patients demonstrated that patients with ICP monitoring were more likely to survive than patients without (OR, 1.40; 95% CI, 1.10-1.78; *P* = .005), and no difference in favorable discharge disposition was found (OR, 0.97; 95% CI, 0.77-1.22; *P* = .77). The E-value for the OR calculated from the matched data set was 1.79. Intracranial pressure monitoring vs no monitoring was associated with an increase in median (IQR) ICU LOS (15 [8.0-21.0] days vs 7 [4.0-12.0] days; *P* < .001) (Table 2). The rate of WOLST was not significantly different between the groups, with a median (IQR) of 6 (4-10) days from presentation (Table 2). No difference in favorable discharge disposition was observed between patients with vs without ICP monitoring (131 [56.2%] vs 135 [57.9%]; *P* = .78) (Table 2).

Given that within the PS-matched data set, GCS score on arrival remained slightly unbalanced, we added GCS score on admission to the logistic regression model. After adjusting for GCS score on admission in the PS-matched data set, ICP monitoring remained associated with survival (OR, 1.61; 95% CI, 1.09-2.39; *P* = .02) and not associated with favorable outcome (OR, 1.08; 95% CI, 0.74-1.56; *P* = .69).

## Discussion

In this comparative effectiveness research study, confounding by indication was addressed using 2 different methods of propensity scoring.^34^ In the matched cohort (466 patients), patients who received ICP monitoring (30.9% mortality) vs patients without monitoring (40.8% mortality) had statistically significant lower mortality on 1:1 PS matching (OR, 1.54; 95% CI, 1.05-2.25) and on PS-weighted analysis (OR, 1.40; 95% CI, 1.10-1.78). Additionally, ICP monitoring was associated with an increased ICU LOS but not with dispositional outcomes.

Analysis of the PS-matched sample attempted to approximate that of an RCT by achieving balance on measured factors between individuals who received ICP monitoring and those who did not. For inverse probability weighting, PSs were used to calculate statistical weights for each individual to create a sample in which the distribution of potential confounding factors was independent of ICP monitoring.^32^ To determine whether the target population that resulted from the application of PS methods was clinically relevant, we emphasized the following inclusion criteria: GCS score under 9 on admission and highest recorded in the first 24 hours, ICU LOS longer than 48 hours (to exclude either mild injuries or early deaths from unsalvageable injuries or WOLST), AIS score of 3 to 5 for the head region and lower than 3 for other body regions (to evaluate patients with relatively isolated head injury), and at least 1 reactive pupil (two-thirds of patients had bilaterally reactive pupils).

In interpreting the results, note that this study used ICP monitoring to distinguish management approaches to patients with monitoring vs those without who were matched for selected clinical characteristics. Information about other treatment interventions was not available. Rather than showing the effectiveness of ICP monitoring, the study results should be interpreted as showing mortality benefit from bundles of care that included ICP monitoring vs no monitoring. It is often cited that civilian PBI has a mortality of up to 90%.^35,36^ This high percentage likely reflects all comers, including patients who either never make it to the hospital or arrive in extremis and die shortly thereafter. In the present study, we excluded such patients. We also excluded patients with bilaterally unreactive pupils and very low values of initial blood pressure and oxygen saturation. These exclusions may explain the mortality rate of 35.8%, which is lower than the rate reported in other contemporary studies. The important message from this finding is that a nihilistic approach toward patients with severe PBI based on the mechanism of injury is not justified, and appropriate resuscitative measures should be undertaken, followed in selected patients by targeted neurocritical and neurosurgical care. This finding was consistent with reports of improved PBI outcomes that were associated with aggressive multidisciplinary care in modern military cohorts.^37,38,39,40^

Prior studies^14,19,41,42^ with mixed results have sought to examine the association between ICP monitoring and LOS in severe blunt TBI. A tendency of extended ICU LOS in patients with ICP monitoring was found in a meta-analysis of 2 RCTs and 7 cohort studies.^43^ Patients with high or low levels of ICP and injury severity will likely experience a shorter ICU stay. This outcome may not be reflected unless an appropriate analysis of time-dependent data (ICP) and event-time data (ICU discharge) is undertaken or jointly modeled.^44^ There are no discharge-time data in the NTDB. Although we matched patients by characteristics that reflected injury severity and rate of WOLST, the analysis cannot offer further explanation of the increased ICU LOS besides the hypothesis that bundles of care including ICP monitoring lead to increased intensity and prolonged courses of treatment.

The Guidelines for the Management of Penetrating Brain Injury was published more than 2 decades ago.^5^ These guidelines found no sufficient data to support any statement about ICP monitoring.^26^ We believe the present study provides moderate quality of evidence in favor of ICP monitoring in select patients with severe PBI. Precision is the degree of certainty surrounding the effect size estimate for a given outcome, and the 2 different methods of propensity scoring we undertook showed similar results. Evidence from this study may provide moderate confidence in the true effectiveness of ICP monitoring in patients with the same characteristics as those of the target population. At a minimum, the results should encourage future RCTs evaluating the efficacy of ICP monitoring in PBI.

### Limitations

This study has several limitations. First, the NTDB does not include data on costs, laboratory values, or long-term functional outcomes^27^; thus, we used dispositional outcome based on discharge location from the hospital. Dispositional outcome should be used only as a rough surrogate of functional outcome at hospital discharge, but no weighty conclusions can be made since discharge location may be affected by socioeconomic factors. Short- and long-term functional outcomes using validated scales, such as the Glasgow Outcome Scale-Extended, needs to be a high priority in future studies of patients with PBI.^45,46^

Second, the PS for each study participant was based on the available measured patient characteristics, and unadjusted confounding may still exist if unmeasured factors changed treatment selection. A meta-analysis by Maragkos et al^47^ on civilian PBI included 17 observational studies (1774 patients) and identified prognostic factors associated with mortality. Increasing age, suicide attempt, lower GCS score, bilateral mydriasis, dural penetration, and bihemispheric and multilobar injury were associated with increased mortality. Although there were no data on injury trajectory in the NTDB, we matched patients for midline shift, highest GCS score, and pupillary reactivity, which represented the closest approximation to overall severity of injury and were potentially associated with missile trajectory. Nevertheless, we cannot exclude unmeasured uncorrelated confounders. For this reason, we performed the sensitivity analysis, which yielded an E-value of 1.79. This E-value meant that the observed OR in favor of ICP monitoring could be explained by an unmeasured confounder that was associated with both ICP monitoring and mortality by an OR of 1.79-fold each, which was above and beyond the measured confounders, but weaker confounding could not do so. Finding such an uncorrelated outcome variable is improbable, given the ORs observed by Deng et al^3^ in their multivariate analysis of factors of death after firearm-related PBI in the NTDB.

## Conclusions

In this comparative effectiveness research study, PBI management that included ICP monitoring was associated with decreased mortality and increased ICU LOS in PS-matched cohorts of patients with severe PBI during the study period (2017-2019). The target population included patients 16 to 60 years of age with an ICU LOS longer than 48 hours, mostly isolated head injury, and at least 1 reactive pupil. Confounding by indication was addressed via a 1:1 PS, PS weighting, and sensitivity analysis. The results, in conjunction with the overall mortality rates and dispositional outcomes observed, challenge prevailing notions of universally poor outcomes after civilian PBI. Aggressive clinical care that includes ICP monitoring may decrease mortality after civilian firearm-inflicted brain injury. Future RCTs that evaluate the efficacy of ICP monitoring in PBI are needed.

## Notes

Supplement 1. **eTable.** ICD-10 Codes

Supplement 2.
Data Sharing Statement
