# Supplementary material for: Comparative Effectiveness of Intracranial Pressure Monitoring vs No Monitoring in Severe Penetrating Brain Injury Management
Source: JAMA Netw Open. 2023 Mar 24;6(3):e231077. doi: 10.1001/jamanetworkopen.2023.1077 (PMC10313150; doi:10.1001/jamanetworkopen.2023.1077)
Supplement: Supplement 1. — eTable. ICD-10 Codes [file jamanetwopen-e231077-s001.pdf]

Supplemental Online Content

Mansour A, Rowell S, Powla PP, Horowitz P, Goldenberg FD, Lazaridis C. Comparative effectiveness of intracranial pressure monitoring vs no monitoring in severe penetrating brain injury management. *JAMA Netw Open*. 2023;6(3):e231077. doi:10.1001/jamanetworkopen.2023.1077

eTable. ICD-10 Codes

This supplementary material has been provided by the authors to give readers additional information about their work.

© 2023 Mansour A et al. *JAMA Network Open.*

**eTable. ICD-10 Codes**	
Code	Description
**S06**	Intracranial Injury
**S06.5**	Traumatic subdural hemorrhage
**S06.6**	Traumatic subarachnoid hemorrhage
**S06.2**	Diffuse traumatic brain injury
**S06.3**	Focal traumatic brain injury
**S06.A**	Traumatic brain compression and herniation
**S06.A0***	Traumatic brain compression without herniation
**S06.A1***	Traumatic brain compression with herniation
**S06.1**	Traumatic brain edema
**S06.4**	Epidural hemorrhage
**S06.8**	Other specified intracranial injuries
**S06.9**	Unspecified intracranial Injury
***Also include ICD S06.A1XA, A1XD, A1XS as well as S06.A0XA, A0XD and A0XS**	

© 2023 Mansour A et al. *JAMA Network Open.*
