# Supplementary material for: Comparative Effectiveness of Intracranial Pressure Monitoring vs No Monitoring in Severe Penetrating Brain Injury Management
Source: JAMA Netw Open. 2023 Mar 24;6(3):e231077. doi: 10.1001/jamanetworkopen.2023.1077 (PMC10313150; doi:10.1001/jamanetworkopen.2023.1077)
Supplement: Supplement 2. — Data Sharing Statement [file jamanetwopen-e231077-s002.pdf]

Data Sharing Statement

Mansour. Comparative Effectiveness of Intracranial Pressure Monitoring vs No Monitoring in Severe Penetrating Brain Injury Management. *JAMA Netw Open*. Published March 03, 2023. doi:10.1001/jamanetworkopen.2023.1077

Data
Data available: Yes
Data types: Data dictionary
How to access data: Data come from the National Trauma Databank; access to the databank requires request and purchase. For our data dictionary email lazaridis@uchicagomedicine.org
When available: With publication

Supporting Documents
Document types: Statistical/analytic code
How to access documents: Requests addressed to lazaridis@uchicagomedicine.org
When available: With publication

Additional Information
Who can access the data: Researchers with approved requests.
Types of analyses: For any purpose.
Mechanisms of data availability: After approval of a proposal.
